# A severe myopathy case in aged patient treated with high statin dosage

**DOI:** 10.1016/j.toxrep.2017.07.009

**Published:** 2017-08-01

**Authors:** Canzonieri Elena, De Candia Cristina, Tarascio Salvatore, Giamporcaro Silvia, Lumera Giovanni, Rigano Giuseppe, Incognito Carmela, Privitera Filippo, Guarnaccia Agata, Foti Pietro Valerio, Palmucci Stefano, Gangemi Pietro, Fuccio-Sanzà Giulia, Signorelli Salvatore Santo

**Affiliations:** a Department of Clinical and Experimental Medicine, University of Catania, Internal Medicine Unit, University Hospital “G.Rodolico”, Catania, Italy; b Radiodiagnostic and Radiotherapy Unit—University Hospital “G.Rodolico ”, Catania, Italy; c Anatomic Pathology Section, University Hospital “Vittorio Emanuele II”, Catania, Italy; d Department of Medical and Surgical Sciences and Advanced Technologies, “G.F. Ingrassia”, Anatomic Pathology Section, University of Catania, Italy

**Keywords:** Statin, Myopathy, Rhabdomyolysis, Enzyme, Inflammatory disease

## Abstract

Statins (S) are widely used drugs for cardiovascular prevention however their utilization may cause a various grade of muscle toxicity. Sometime S discontinuation alone is not sufficient to revert muscle injury and this can evolve in serious inflammatory muscle disease. In this case immunosuppressive medications are required to achieve remission. This case report describes a patient who developed rhabdomyolysis after recent S treatment initiation and the diagnostic work up have lead to the diagnosis of necrotizing autoimmune myopathy (NAM). We believe that the clinical case described here is a useful report of this rare toxicity and we aim to highlight the importance of its prompt recognition and treatment.

## 1 Introduction

The benefits of statins for secondary and primary prevention in people at moderate and high risk of cardiovascular disease are undisputed [1]. Despite these drugs are deemed to have a favorable safety profile, no drug is without potential for adverse events (AEs) [2]. Muscle AEs are the most common toxicity associated with statin use and clinically this can include muscle pain, fatigue and weakness up to rhabdomyolysis [2]. However, a large number of patients are asymptomatic and only present an increase in hepatic and muscle enzyme, particularly creatine kinase (CK). The most common musculoskeletal disorder is myalgia (5% of patients). Myopathy is developed in 0.1–0.2% patients, and rhabdomyolysis (Rhab) in 0.01% patients [2]. Usually, symptoms and laboratory alterations disappear after S discontinuation but rarely can evolve in serious inflammatory muscle involvement [3]. In the latter case patients may require immunosuppressive therapy (e.g. corticosteroid, immunoglobulin, azathioprine, mycophenolate mofetil) to control the inflammatory response and revert the clinical scenario but, unfortunately, relapse can occur while tapering the immunosuppressive medication [4]. In this case report, we describe the case of a patient who developed Rhab due to S use, and we believe that the reported clinical case is a useful representation of this unusual disease.

## 2 Case history, discussion and conclusion

A 82-year-old man was admitted to our GIM Unit (General Internal Medicine, University Hospital G.Rodolico, Catania, Italy) because of the gradual onset of asthenia, muscular pain, tenderness and decreased muscle strength in the previous two weeks.

The patient past medical history was notable for arterial hypertension, prediabetes, dyslipidemia, and chronic renal failure. Moreover, three weeks before being hospitalized the patient had suffered an acute anterior-septal myocardial infarction, treated with primary percutaneous coronary intervention and stenting. At that time, the patient was started on beta blocker, calcium antagonist, antianginal (ranolazine 375 mg b/die), S (atorvastatin 80 mg b.p.d.), antiplatelet (aspirin and ticagrelor), and ursodesossicolic acid.

Upon admission to our Unit vital signs were normal (arterial blood pressure: 115/80 mmHg, heart rate: 72 bts/min.) A 12-Lead electrocardiogram showed regular sinus rhythm with defects in the repolarization phase of the anterior-septal derivations due to recent myocardial infarction.

Laboratory test at admission (Table 1) showed high level of creatinine 2.67 mg/dl, CK >3145 U/L, Lactate Dehyidrogenase (LDH) 262 U/L. After six hours we observed a further increase in muscle enzymes values: CK 5533 U/L, mass-CK 19.40 ng/mL, Troponine I HS 209 ng/L, LDH 273 U/L, and hepatic enzyme (aspartate aminotraferase, AST) up to 46 U/L. Based on patient’s symptoms and laboratory findings we diagnosed Rhab. Within the course of the subsequent 24 h we first suspended atorvastatin and then ranolazine; continuous saline infusion was started with diuresis and volume status monitoring.

**Table 1:** Patient’s laboratory test at the time of admission.

Test	Value	Test	Value	Test	Value
Haemoglobin (g/dl)	10.9	Calcium mg/dL	7.8	Total bilirubine (mg/dL)	1.4
Red cells (mm^3^)	4.83 106/μL	Urico acid (mg/dL)	2.8	Lactive deidrogenase (LDH) U/L	262
White cells(mm^3^)	11.33	Creatinphosphokinase (CPK) U/L	3145	CRP (mg/L)	26.7
Platelet (μL)	280.000	Proteines (g/dL)	4.40	Eritrocithary sedimentation velocity (EVS) mm	45
Creatinine (mg/d)L	2.67	Albumin (g/dL)	2.14	Fibrinogen (mg/dL)	560
Glomerural filtratiorate rate mL/min	21	Total cholesterol mg/dL	103	Thrombin time (TIP) seconds	30
Na (mmol/l)	135	Liver enzymes (U/L)	-/37	Thrombin time (INR)	0.87
K (mmol/l)	4.2	Gamma glutamil transferase (γ-GT) U/L	45/17	Urine	Hb + ++

Interestingly, Ranolazine has hepatic metabolism mainly by CYP3A. It presents drug interaction with S (substrate of CYP3A). Ranolazine shows 1.3 fold increases in both maximal concentration (Cmax) and area under curve (AUC) of distribution of S (atorvastatin), and also reduces by up to 35% Cmax and AUC of the drug metabolites.

Despite discontinuing S and continuous saline infusion, CK values significantly rose to 124095 U/L. The others lab test increased as follows: LDH 2105 U/L, AST 1521 U/L, ALT 343 U/L, myoglobin 4000 ng/mL, and serum creatinine 2.79 mg/dL (Table 2).

**Table 2:** Progressive increase of patient’s laboratory values over time.

Day	08.02.17	08.02.17	09.02.17	09.02.17	10.02.17	11.02.17	11.02.17
Time	h 8.00	h 18.00	h 8.00	h 15.00	h 8.00	h 8.00	h 16.00
Test	Value						
CPK (U/L)	8080	15439	24803	27021	35703	111660	124095
Mioglobin (ng/ml)	>4009				>4009	>4009	
CK-mass (ng/ml)	20	33.90	40.30		53	127	
Troponine (ng/L)		156			91	214	
Creatinine mg/dL	2.96	3.12	2.96	2.95	2.96	2.79	
LDH (U/L)		381	535	519	681	2105	2141
AST/ALT (U/L)		-/82	485/113	-/128	699/179	1521/343	-/413

Similar clinical case defined as S induced Rhab have been recently published and are clinically characterized by: muscle weakness, high CK enzyme levels, and importantly lack of improvement after statin discontinuation [4]. In 2012, Padalaa and Thompson conducted a review of the literature and found twenty-four out of sixty-three cases that could be identified as S-related autoimmune myophaty. The recommended treatment in these cases is immunosuppressive therapy [5].

Therefore the patient was started on methylprednisolone 1 mg/kg/day and we observed a gradual decrease of muscle enzymes values and their complete normalization after 10 days of treatment along with the resolution of both weakness and muscle pain.

**Fig. 1:**
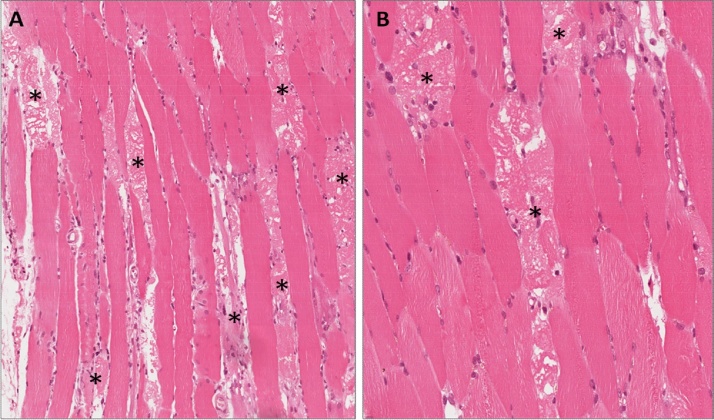
Incisional biopsy from skeletal muscle of the buttock. (A) Low magnification showing necrosis (*) of some skeletal muscle cells. (B) Higher magnification showing necrotic cells (*) scattered among normal-appearing skeletal muscle cells. Inflammatory infiltrate and fibrosis are lacking.

**Fig. 2:**
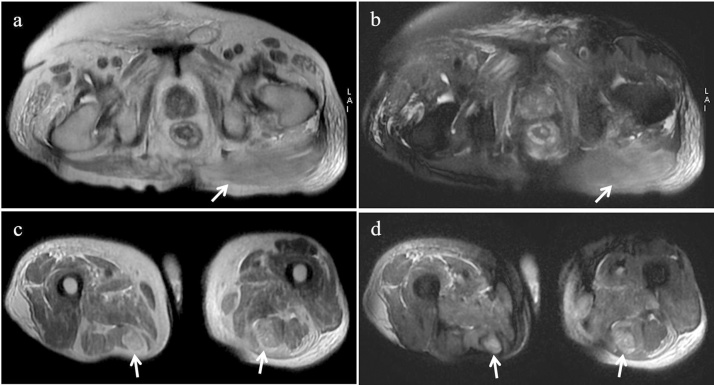
Muscle oedema on fluid-sensitive MRI sequences. Axial T2-weighted **(a)** and fat-suppressed T2-weighted **(b)** MR images show diffuse increased signal intensity of the left gluteus maximum muscle (white arrows). Axial T2-weighted **(c)** and fat-suppressed T2-weighted **(d**) MR images show atrophy and increased signal intensity of the right and left semitendinosus muscles (white arrows).

The electromyography test of the peripheral muscles revealed a lack of spontaneous muscle activity at rest. The test showed reduction of electrical activity at early recruitment due to an acute muscle damage across all the examined muscles. Gluteus muscle biopsies revealed lytic necrosis of the myocytes at different stages, and significant macrophage infiltration with limited presence of TCD4+ cells (Fig. 1). Muscle bilateral thighs Magnetic Resonance (Fig. 2) found widespread hyperintensity and oedema of gluteus muscle. Similar findings were found for other muscles of the lower limb (semi-tendon muscles of both lower legs).

Necrotizing autoimmune myopathy (NAM) is part of a wide group of inflammatory myopathies that include also polymyosite and dermatomyosite [6]. Many studies showed a strong association between exposure to S (atorvastatin, simvastatin, pravastatin and rosuvastatin) and the development of NAM [7], [8]. This data implies that this is a class effect and not a specific statin effect, however more research is needed to arrive at an indisputable conclusion.

Similar to more common S associated myopathies, the clinical presentation of NAM is characterized by sub-acute and progressive weakness of the lower limb proximal muscles associated with an increase of CK blood levels. However NAM does not resolve with S discontinuation and is actually associated with a progressive worsening myositis that requires immunosuppression. The relapse rate is high after drug tapering [7].

Autoantibodies against 3-hydroxy-3-methylglutaryl-coenzyme A reductase (HMGCR) have been identified in patients with NAM and they are detected through ELISA. HMGCR is a key enzyme in the cholesterol biosynthesis pathway and it is inhibited by statin. Several studies have established that anti-HMGCR antibodies are specifically detected in immunological myopathy induced by statins [8]. In our hospital, antibody measurements have not been reliable so unfortunately we could not make any autoantibody remarks.

NAM is a necrotizing myopathy with marked myofiber degeneration, abundant macrophages, and minimal lymphocytic infiltrate [9] thus differing form polymyositis and dermatomyositis muscle biopsies that have a different histological pattern. In this case, the biopsy findings are consistent with NAM.

Immunosuppressive drugs (i.e. corticosteroid, immunoglobulins, azathioprine, and micophenolate) are effective in reducing the autoimmune aggression to muscular tissues. However, combination therapy may be advisable as there is a high risk of relapse after tapering or discontinuation of therapy.

The patient had a brilliant response to high dose corticosteroids (regression of spontaneous pain and weakness, and normalization of enzyme levels), but unfortunately he was transferred to another Unit soon after the resolution of the acute phase and we have no information on the steroids tapering phase and other general follow up data.

We believe that this case report is representative of a very rare adverse event associated with statin use and highlights the importance of a prompt recognition and immunosuppressive treatment start to obtain a favorable outcome.

The mechanisms at the origin of NAM are unclear and future studies must be performed to analyze specific etiologies and recognize precocious markers of NAM. In this way, we could identify patients at risk, evaluate best the costs and benefits related to S and pay more attention to choosing the molecules and their dosages.
