# Supplementary material for: A severe myopathy case in aged patient treated with high statin dosage
Source: Toxicol Rep. 2017 Aug 1;4:438–40. doi: 10.1016/j.toxrep.2017.07.009 (PMC5615159; doi:10.1016/j.toxrep.2017.07.009)
Supplement: Supplementary file 2 [file mmc2.docx]

**MethodsX article template**

***GENERAL INFORMATION***

Article focuses on the damage on metabolic pathway (liver,muscle) originated by anti lipemic drug. This deleterious effect should be immediately evident to the reader.

**Title: A severe myopathy case in aged patient treated with high statin dosage.**

**Authors:** Canzonieri Elena^1^, De Candia Cristina^1^, Tarascio Salvatore^1^, Giamporcaro Silvia^1^, Lumera Giovanni^1^, Rigano Giuseppe^1^, Incognito Carmela^1^, Privitera Filippo^1^, Guarnaccia Agata^1^, Foti Pietro Valerio^2^, Palmucci Stefano^2^, Gangemi Pietro**^3^,** Fuccio-Sanzà Giulia^4^, Signorelli Salvatore Santo. ^1^

**Affiliations:** ^1^Department of Clinical and Experimental Medicine. University of Catania. Internal Medicine Unit. University Hospital “G.Rodolico”. Catania (Italy)^2^Radiodiagnostic and Radiotherapy Unit – University Hospital “G.Rodolico ”, Catania, (Italy)^3^ Anatomic Pathology Section, University Hospital “Vittorio Emanuele II”. Catania, (Italy)^4^Department of Medical andSurgical Sciences and Advanced Technologies, "G.F. Ingrassia", Anatomic Pathology Section, University of Catania. (Italy)

**Contact email:** **ssignore@unict.it;antonella1signorelli@gmail.com***]*

**Abstract:** Statins (S) are widely used drugs for cardiovascular prevention however their utilization may cause a various grade of muscle toxicity. Sometime S discontinuation alone is not sufficient to revert muscle injury and this can evolve in serious inflammatory muscle disease. In this case immunosuppressive medications are required to achieve remission. This case report describes a patient who developed rhabdomyolysis after recent S treatment initiation and the diagnostic work up have lead to the diagnosis of necrotizing autoimmune myopathy (NAM). We believe that the clinical case described here is a useful report of this rare toxicity and we aim to highlight the importance of its prompt recognition and treatment.

**Keywords:** Statin, Myopathy, Rhabdomyolysis ,Enzyme, Inflammatory disease

**Specifications Table**

| Subject area | *Select one of the following subject areas:*   - *Agricultural and Biological Sciences* - *Biochemistry, Genetics and Molecular Biology* - *Chemical Engineering* - *Chemistry* - *Computer Science* - *Earth and Planetary Sciences* - *Energy* - *Engineering* - *Environmental Science* - *Immunology and Microbiology* - *Materials Science* - *Mathematics* - *Medicine and Dentistry* - *Neuroscience* - *Pharmacology, Toxicology and Pharmaceutical Science* - *Physics and Astronomy* - *Psychology* - *Social Sciences* - *Veterinary Science and Veterinary Medicine* |
| --- | --- |
| More specific subject area | *Drus and metabolismDescribe narrower subject area* |
| Method name | *Case report* |
| Name and reference of original method | *no* |
| Resource availability | *no* |

**Method details** *Authors show clinical situation occurred.*

**Acknowledgements:** *None*

**Supplementary material *and/or* Additional information:** *None*

**References:** P. Mohassel and A. L. Mammen, “Statin-associated autoimmune myopathy and anti-HMGCR autoantibodies,” *Muscle and Nerve*, vol. 48, no. 4, pp. 477–483, 2013.

M. Klein, H. Mann, L. Pleštilová, J. Zámečník, Z. Betteridge, N. McHugh, and J. Vencovský, “Increasing incidence of immune-mediated necrotizing myopathy: single-centre experience,” *Rheumatology (Oxford).*, vol. 54, no. 11, pp. 2010–2014, 2015.

D. Kassardjian, V. A. Lennon, N. B. Alfugham, M. Mahler, and M. Milone, “Clinical Features and Treatment Outcomes of Necrotizing Autoimmune Myopathy,” *JAMA Neurol.*, vol. 72, no. 9, p. 996, 2015.
